# Reliability of patient-specific gait profiles with inertial measurement units during the 2-min walk test in incomplete spinal cord injury

**DOI:** 10.1038/s41598-024-53301-y

**Published:** 2024-02-06

**Authors:** Romina Willi, Charlotte Werner, László Demkó, Rob de Bie, Linard Filli, Björn Zörner, Armin Curt, Marc Bolliger

**Affiliations:** 1 Spinal Cord Injury Centre Balgrist, University Hospital, Zurich, Switzerland; 2 Department of Epidemiology, Maastricht University, Maastricht, The Netherlands; 3 Swiss Center for Movement Analysis (SCMA), Balgrist Campus AG, Zurich, Switzerland

**Keywords:** Rehabilitation, Movement disorders

## Abstract

Most established clinical walking tests assess specific aspects of movement function (velocity, endurance, etc.) but are generally unable to determine specific biomechanical or neurological deficits that limit an individual’s ability to walk. Recently, inertial measurement units (IMU) have been used to collect objective kinematic data for gait analysis and could be a valuable extension for clinical assessments (e.g., functional walking measures). This study assesses the reliability of an IMU-based overground gait analysis during the 2-min walk test (2mWT) in individuals with spinal cord injury (SCI). Furthermore, the study elaborates on the capability of IMUs to distinguish between different gait characteristics in individuals with SCI. Twenty-six individuals (aged 22–79) with acute or chronic SCI (AIS: C and D) completed the 2mWT with IMUs attached above each ankle on 2 test days, separated by 1 to 7 days. The IMU-based gait analysis showed good to excellent test–retest reliability (ICC: 0.77–0.99) for all gait parameters. Gait profiles remained stable between two measurements. Sensor-based gait profiling was able to reveal patient-specific gait impairments even in individuals with the same walking performance in the 2mWT. IMUs are a valuable add-on to clinical gait assessments and deliver reliable information on detailed gait pathologies in individuals with SCI.

**Trial registration:** NCT04555759.

## Introduction

Gait impairments are prevalent among individuals with neurological disorders^1^, affecting more than 60% of these patients^2^. These impairments often show common gait abnormalities, such as slower gait speed, shorter stride length, and poor body balance^3^. Given that gait function is integral to daily life activities^4^, disturbances in gait often represent the initial stages towards loss of mobility and independence^2,5^. As such valid, reliable, and sensitive assessments of walking function are crucial in quantifying gait impairments in individuals with neurological disorders, thereby enabling these impairments to be therapeutically addressed.

A variety of clinical tests exist to assess walking function. They range from assessments of walking ability during different activities to timed measures determining how many meters one can walk in a given time or how long it takes to walk a given distance. Each of these measures has specific advantages and limitations. However, gait speed (derived from timed measures) is probably the most established clinical outcome to monitor walking function, as it can be considered as a key feature of locomotor control and is easy to assess in clinical practice^6^. The main drawback of timed measures is their inability to assess specific individual deficits or gait characteristics underlying a gait impairment.

Locomotion is controlled at multiple levels of the central nervous system (CNS), i.e. cortical, subcortical, brainstem and spinal networks^7,8^. Various domains, such as pace, rhythm, variability, asymmetry, postural control, etc.^9–11^ can partly describe these physiological systems. Assessing these different domains of locomotor function by gait analysis allows the construction of individual gait profiles that are unique to each person, similar to “fingerprints”, particularly after spinal cord injury (SCI)^9^. For gait analysis, optical motion capture (OMC) systems are considered the gold standard^12^. However, these systems come with the disadvantages of high cost, the need for expert operation, time-consuming setup and the fact that it is restricted to a laboratory setting^13^, which does not make them practical for everyday clinical use.

In recent years, inertial measurement units (IMU) have been used as a possible alternative to objectively assess gait^14^ and could be a valuable extension of standard functional gait tests. Compared to OMC, IMUs are compact, inexpensive, easy to operate and can be combined with functional walking tests, making them convenient for clinical applications or gait assessments even in the home environment^13^. Even a sparse IMU setup of only two IMUs attached to the lower extremities can provide reliable spatiotemporal gait parameters in individuals without neurological movement disorders^15^. In individuals with mild walking impairments, sensor setups have been developed that show high validity and good test–retest reliability in several neurological diseases^16–18^. However, a previous study has shown that the detection of gait events by such IMU setups is unreliable in individuals with slow walking speeds (< 1.2 m/s) and short stride lengths (< 1.0 m)^19^, often observed in individuals with severe gait impairments. A new sensor setup and algorithm for IMU-based gait analysis (ZurichMOVE, https://zurichmove.com) has recently been validated in individuals with severe SCI, walking at slow speeds (mean walking speed of 0.76 ± 0.17 m/s) in a laboratory environment^20^. The IMU modules consist of a tri-axial accelerometer, a tri-axial gyroscope, and a tri-axial magnetometer that record at a sampling rate of 200 Hz. Due to indoor magnetic field distortions, the magnetometer data was excluded from the data analysis^20^. The setup involves two sensors attached laterally just above each ankle, and the developed algorithms provide valid spatiotemporal parameters even for individuals with severe walking impairments walking at very low speeds. Using such IMU setups in combination with timed measures would allow us to understand individuals’ gait characteristics and interpret mechanisms that lead to changes in gait performance over consecutive assessments. Nevertheless, prior to combining an IMU-setup and the implemented algorithms with a timed measure like the 2mWT, their reliability must be established.

This study aimed to investigate whether the ZurichMOVE sensor setup and the newly developed algorithms can reliably assess gait characteristics of individuals with SCI performing a 2mWT. Firstly, test–retest reliability of the sensor setup was evaluated in two measurements performed within a week. Subsequently, we describe whether the setup is sufficiently sensitive to discern various gait profiles among individuals with comparable walking performance.

## Methods

### Participants

Individuals with SCI were recruited at the Spinal Cord Injury Center of the Balgrist University Hospital. The inclusion criteria were acute/subacute (1–6 months) or chronic (> 6 months) SCI, age ≥ 18 years and able to walk at least 10 m with/without braces, assistive devices at a minimum walking speed of 0.17 m/s.

The exclusion criteria were current orthopaedic issues, major psychosis, depression, a history of severe heart conditions, and other neurological diseases.

### Procedure

The study has been approved by the Ethical Committee of the Canton of Zurich (BASEC 2020-01473) and was conducted in accordance with Good Clinical Practice (GCP) guidelines and the Declaration of Helsinki. Prior to enrolment, written informed consent has been obtained from all participants. Individuals were invited to participate on 2 test days, separated by 1 to 7 days. On the first day, a familiarisation run of the 2-min walk test (2mWT) was performed. After a break of at least 30 min, another 2mWT was performed. On the second test day, the 2mWT was repeated. In addition, the lower extremity motor score (LEMS) was assessed on the first day.

### Sensor setup

**Figure 1:**
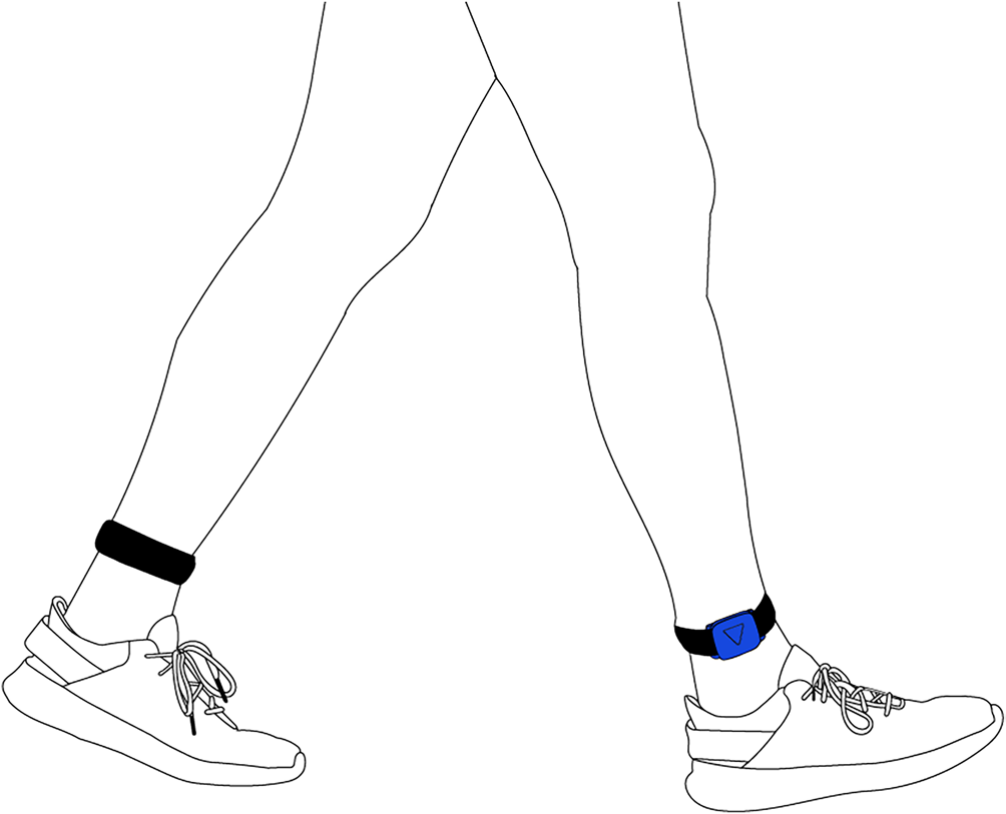
Positions of the sensors attached laterally above the ankles.

Spatiotemporal gait parameters were assessed by two IMU sensors (ZurichMOVE, https://zurichmove.com) (measuring 35 × 35 × 12 mm and weighing 18 g)^21^. More details on the IMU sensor modules are described in a previous study^21^. They were attached to the ankles just above the lateral malleolus with an elastic Velcro strap (Fig. 1).

### Assessment of gait profiles

Gait profiles were created consisting of sensor-derived parameters that could be divided into spatiotemporal measures (gait cycle parameters expressed as mean of multiple strides) and dynamic features (representing the inconsistency of spatiotemporal measures across the strides^20^. Spatiotemporal gait parameters except smoothness were calculated using a previously developed algorithm^20^ and they were extracted for each cycle, namely: stride velocity (m/s), stride time (s), step time (s), swing time (s), relative swing time (%), stance time (s), relative stance time (%), double support (s), relative double support (%), stride length (cm), horizontal foot displacement in frontal plane (cm), vertical foot displacement in sagittal plane (cm) and smoothness (see Supplementary Table 1 for the detailed description of each parameter). The algorithm identified turning steps as steps that deviated more than 30° from the main movement direction. One step before the turn, the steps taken during the turn, and one step immediately after the turn were excluded from the analysis. Smoothness was calculated based on analytical models from Balasubramanian et al.^22^. Dynamic features of gait were deduced by calculating symmetry and variability parameters from all sensor-derived spatiotemporal parameters. Symmetry (symmetry index: $SI= \frac{L-R}{max}\times 100$)^23^ and variability ((coefficient of variance: $COV= \frac{\sigma }{\mu }$), σ = standard deviation; μ = mean)) were calculated. Gait parameters were grouped in functional domains according to the literature^9,10,24,25^. This resulted in five gait domains with the corresponding parameters defined in Table 1. To generate a visual representation of the gait profiles, we normalized the data by converting them into z-scores, using the data of the first day as a basis for this transformation. During this process, for each subject, we calculated the mean values across all their gait cycles, then used these z-scores to create a radar graph, providing a clear visual depiction of the data.

**Table 1:** Allocation of gait parameters into gait domains.

Domain	Parameters
Pace	Stride length, swing time variability and stride velocity
Variability	Step time variability, stance time variability, stride velocity variability, stride length variability
Rhythm	Step time, stance time, swing time
Asymmetry	Step time asymmetry, stance time asymmetry and swing time asymmetry
Postural control	Stride length asymmetry, double support

Domains were adapted from Refs.^9,10,24,25^.

### 2-min walk test

The 2mWT was performed according to the Guidelines of the American Thoracic Society^26^, except that the hallway length was 35 m (instead of 30 m). The decision to alter the length was prompted by the narrowness of the corridor. By extending it to a length of 35 m, more space was available for the turns. For safety reasons, individuals were accompanied by an examiner walking behind each individual to allow them to set the pace.

Braces and/or habitual assistive devices were permitted but must be kept similar across the assessments. The individuals were instructed to walk as far as possible but safely within two minutes. They were allowed to take rest breaks if needed, but time continued running during the break.

The participants were asked to wear closed, comfortable shoes. Shoes with high heels (> 3 cm) were excluded. Shoes were identical for both assessments.

### Statistics

#### Sample size calculation

Sample size calculation for the assessment of test–retest reliability was conducted using the formula proposed by Walter et al.^19^. The formula was implemented in an online calculator (https://wnarifin.github.io/ssc/ssicc.html). The sensor-derived parameters were validated with an infrared marker-based motion capture system^20^ which is considered the gold standard in modern gait analysis due to their high level of precision^27^ Based on the high accuracy of the sensor-derived parameters as demonstrated in this validation study, we anticipated that the true intraclass correlation coefficients (ICC) would be as well excellent with a value of approximately 0.95, and a minimal acceptable ICC set at 0.85. The significance level was chosen as 0.05 at a power of 0.8. Repetitions were set to 2. Based on these considerations, the minimal required sample size for the study was determined to be n = 25.

#### Test–retest reliability

ICCs (two-way mixed effect, total agreement) were calculated to determine test–retest reliability of all parameters between the first and second day^28^. ICC values have been interpreted according to the recommendation of Koo et al.^29^: < 0.5: “poor”, 0.5–0.74: “moderate”, 0.75–0.9 “good”, and > 0.9 “excellent”. Bland–Altman plots have been created to estimate the absolute agreement between the two measurements.

The standard error of measurement (($SEM=SD\times \sqrt{(1-ICC)}$), SD = standard deviation)^30^ and the Minimal Detectable Change ($MDC=SEM\times 1.96\times \sqrt{2}$)^31^ were calculated based on the respective formulas.

#### Consistency of gait profiles

To assess the consistency of the gait profiles between days 1 and 2, the sum of the absolute z-score values was calculated and then compared using a t-Test.

### Ethical approval

The Ethics Committee of the Canton of Zurich (BASEC 2020-01473) approved this study. All participants gave written informed consent before data collection began.

## Results

### Participant characteristics

Twenty-eight individuals with moderate to severe gait impairments have been recruited for this study and assessed. Data of two individuals had to be excluded from the analysis due to technical problems with the sensors. Data of 26 individuals (age: 57.1 ± 14.6) with SCI (AIS C: 7; AIS D: 19) were analysed in this study. Further demographic characteristics of the study participants are depicted in Table 2.

**Table 2:** Detailed demographic characteristics of the individuals with spinal cord injury who participated in the study.

Variable	Value
Age (year)	57.1 ± 14.6
Sex	
Male	16 (62)
Female	10 (38)
Height (cm)	174.4 ± 10.2
Weight (kg)	76.4 ± 18.5
BMI (kg/m^2^)	25.0 ± 4.6
NLI	
Tetraplegic	14 (54)
Paraplegic	12 (46)
AIS-grade	
C	7 (27)
D	19 (73)
Type of injury	
Traumatic	14 (54)
Non-traumatic	12 (46)
LEMS (max score 50)	43 ± 16
Years since injury	5.0 ± 5.8
WISCI II (range)	8–20
Distance 2mWT (range) (m)	20–241

Data are presented as mean ± SD or number (percentage).

*NLI* neurological level of injury, *AIS* American Spinal Injury Association Impairment Scale, *LEMS* lower extremity motor score, *WISCI II* walking index for spinal cord injury II.

### Test–retest reliability

**Figure 2:**
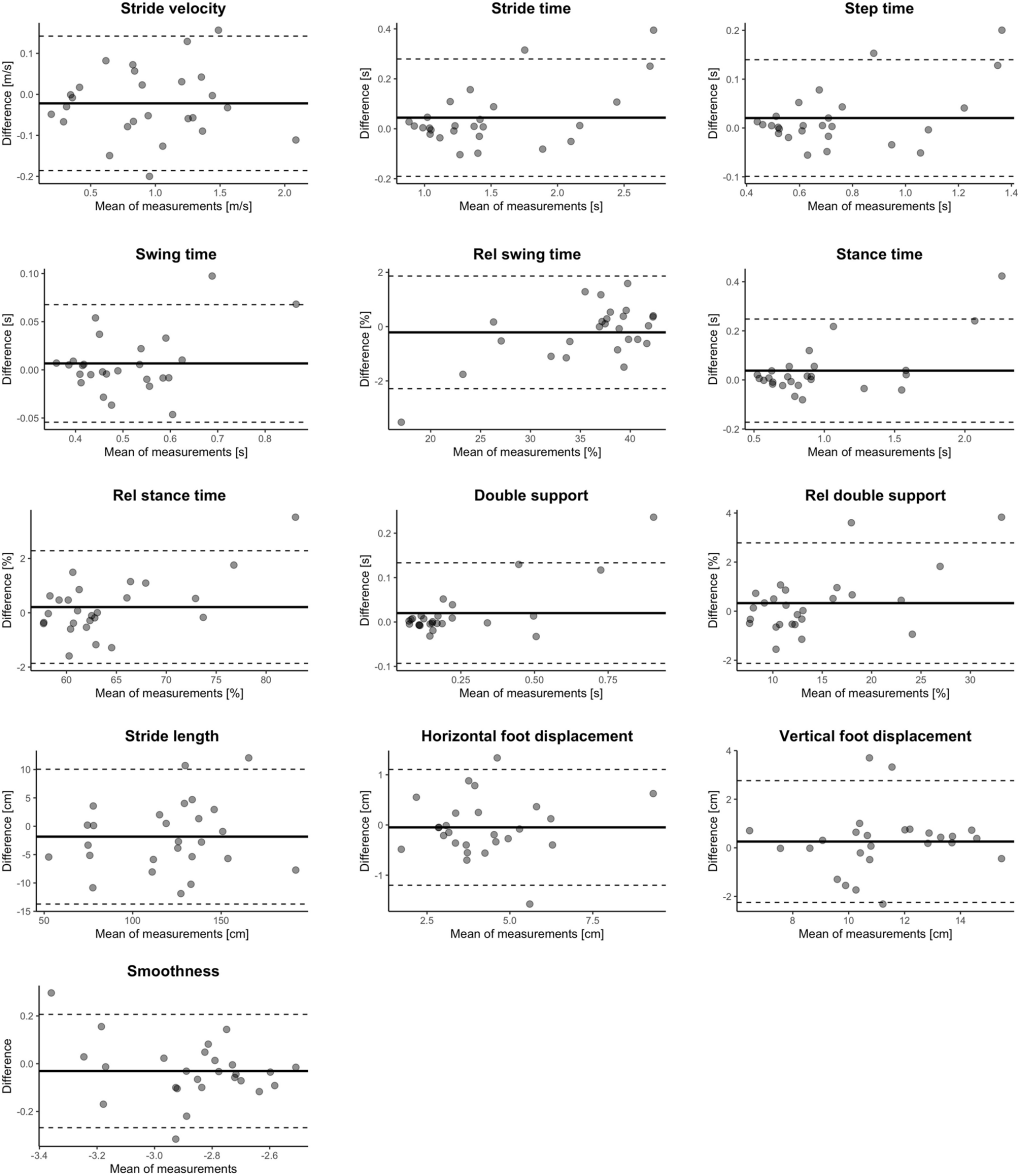
Bland–Altman plots to assess test–retest reliability. The averages of the two measurements were plotted against the differences. The solid lines represent the means and the dashed lines the 95% limits of agreement.

In total, 8608 strides of 26 individuals with SCI were included in the analysis. The mean distance in the 2mWT did not differ between the test days (p = 0.086) and was 105 m ± 54 m (min–max: 20–234 m) on the first day and 109 m ± 54 m (min–max: 25–241 m) on the second day. On average, the testing days were spaced 3.6 ± 1.65 days apart. The number of strides did not differ between the tests (p = 0.322), with an average of 165 strides (min–max: 79–252) in the first and 166 strides (min–max: 79–258) in the second 2mWT. The gait parameters showed good to excellent ICCs (0.84–0.99 for spatiotemporal measures and 0.77–0.99 for dynamic measures). More details about the averaged gait parameters, ICCs, SEM and MDC can be found in Table 3. No significant difference in any parameters was found between days 1 and 2. The Bland–Altman plots of the parameters showed a reasonable agreement between the days (Fig. 2).

**Table 3:** Test–retest reliability and minimal detectable change of spatiotemporal parameters.

Parameter	Mean ± SD Day1	Mean ± SD Day2	ICC	Agreement interpretation	SEM	MDC
Stride velocity (m/s)	0.94 ± 0.48	0.96 ± 0.47	0.98 (0.965–0.993)	Excellent	0.07	0.20
Stride time (s)	1.51 ± 0.57	1.46 ± 0.51	0.97 (0.938–0.988)	Excellent	0.08	0.23
Step time (s)	0.75 ± 0.29	0.73 ± 0.25	0.97 (0.939–0.988)	Excellent	0.04	0.12
Swing time (s)	0.51 ± 0.12	0.50 ± 0.11	0.96 (0.918–0.983)	Excellent	0.02	0.06
Relative swing time (%)	35.94 ± 6.60	36.15 ± 5.95	0.99 (0.969–0.994)	Excellent	0.70	1.95
Stance time (s)	1.00 ± 0.50	0.96 ± 0.43	0.97 (0.935–0.987)	Excellent	0.07	0.20
Relative stance time (%)	64.06 ± 6.60	63.85 ± 5.95	0.99 (0.969–0.994)	Excellent	0.70	1.95
Double support (s)	0.25 ± 0.23	0.23 ± 0.19	0.96 (0.911–0.982)	Excellent	0.04	0.11
Relative double support (%)	14.28 ± 6.77	13.95 ± 6.06	0.98 (0.957–0.991)	Excellent	0.86	2.37
Stride length (cm)	117.92 ± 33.88	119.76 ± 32.94	0.98 (0.962–0.992)	Excellent	4.29	11.90
Horizontal foot displacement (cm)	4.20 ± 1.62	4.25 ± 1.57	0.93 (0.859–0.970)	Excellent	0.40	1.10
Vertical foot displacement (cm)	11.41 ± 2.33	11.15 ± 2.22	0.84 (0.681–0.925)	Good	0.88	2.44
Smoothness	− 2.88 ± 0.21	− 2.85 ± 0.24	0.85 (0.696–0.929)	Good	0.09	0.26
Step time asymmetry (%)	− 1.62 ± 17.82	1–1.73 ± 17.01	0.99 (0.981–0.996)	Excellent	1.61	4.47
Swing time asymmetry (%)	− 1.74 ± 16.61	− 1.64 ± 16.00	0.98 (0.957–0.991)	Excellent	2.21	6.11
Stance time asymmetry (%)	1.92 ± 9.33	1.56 ± 8.92	0.98 (0.951–0.990)	Excellent	1.32	3.67
Stride length asymmetry (%)	0.32 ± 5.35	− 0.11 ± 5.23	0.77 (0.545–0.888)	Good	2.53	7.01
Stride length variability	0.08 ± 0.03	0.08 ± 0.04	0.79 (0.583–0.900)	Good	0.02	0.05
Stride velocity variability	0.10 ± 0.04	0.10 ± 0.04	0.87 (0.72–0.939)	Good	0.02	0.04
Stance time variability	0.08 ± 0.04	0.07 ± 0.03	0.85 (0.695–0.31)	Good	0.01	0.03
Step time variability	0.10 ± 0.09	0.10 ± 0.08	0.97 (0.931–0.986)	Excellent	0.02	0.04
Swing time variability	0.10 ± 0.07	0.10 ± 0.06	0.97 (0.926–0.985)	Excellent	0.01	0.03

Data are presented as mean ± SD or number, 95%-confidence intervals in parentheses.

*ICC* intra-class correlation, *MDC* minimum detectable change, *SEM* standard measurement error.

The Bland Altman plots of the dynamic parameters can be found in Supplementary Fig. 1.

### Consistency of gait profiles

**Figure 3:**
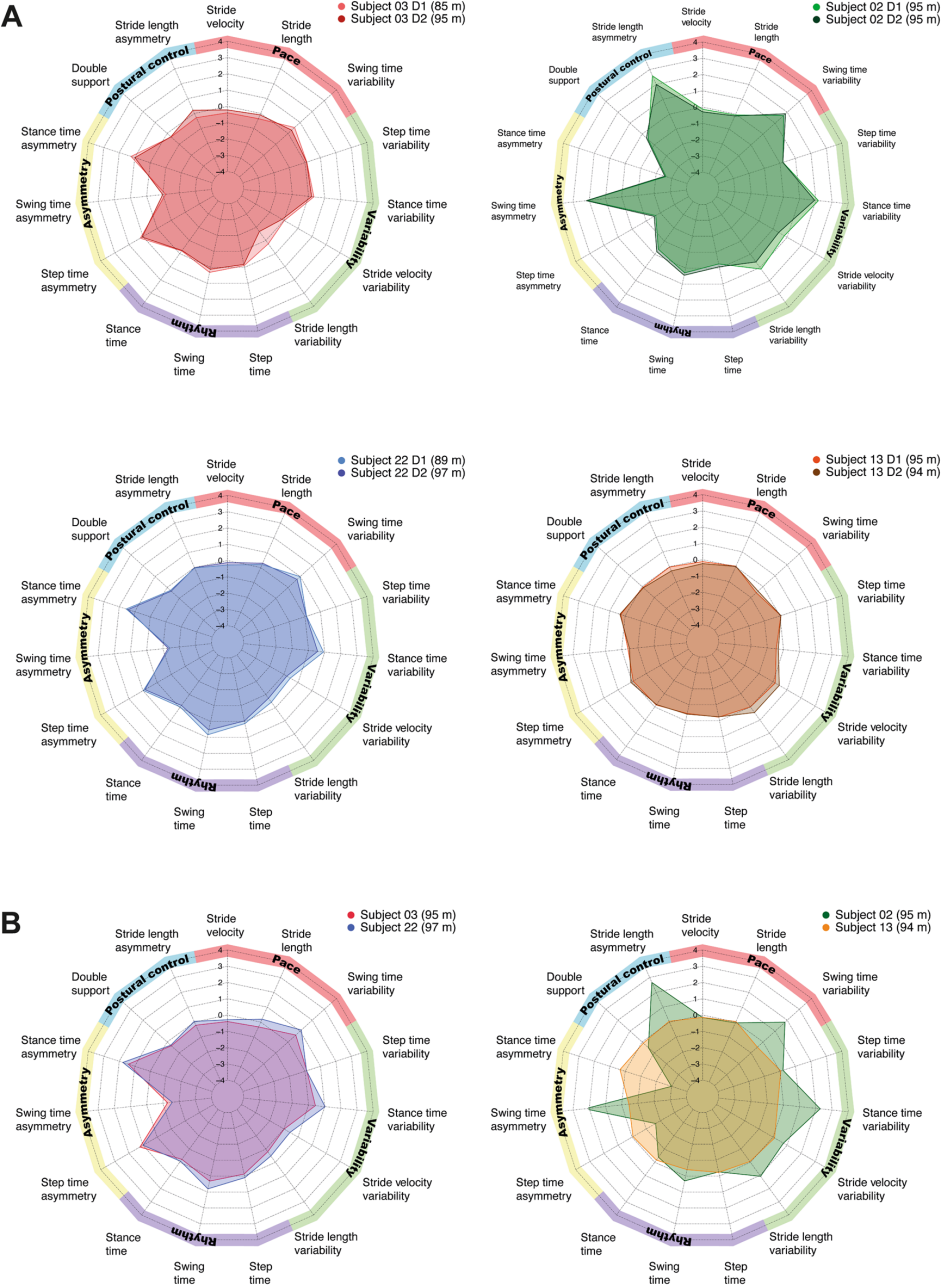
Radar chart illustrating the 15 gait characteristics organised by domains. (**A**) Shows the consistency of the gait profile from day 1 (D1) to day 2 (D2) for four individuals with a similar performance in the 2-min walk test. (**B**) Shows different gait strategies used by individuals, albeit same walking distances. Two show similar gait profiles (right subplot) and two show profoundly different gait profiles (left subplot).

The comparison of the absolute summed z-scores between day 1 (11.07 ± 6.13) and day 2 (10.37 ± 5.1) revealed no significant difference (p = 0.1228). Figure 3A shows the gait profile of 4 individuals who covered almost the same distance in the 2mWT. All four individuals show a similar profile on both testing days, illustrating the within subject consistency of the gait profiles. However, gait profiles also illustrate that gait characteristics may differ between individuals with comparable walking distance in the 2mWT (Fig. 3B). This is in accordance with the substantial left–right asymmetry in the LEMS (left: 10, right: 25) found for subject 02, whereas subject 13 achieved the maximal score of 25 with both legs.

The z-scores for all individuals, calculated for day 2 are presented in Supplementary Table 2.

## Discussion

Gait impairments are frequently seen in neurological diseases. Therefore, it is essential to objectively assess walking impairments in order to offer appropriate physical therapy interventions, measure the success of treatments, and monitor disease progression.

The present study aimed to assess the reliability of IMU-based gait parameters in individuals with SCI. The main findings of the present study are that in individuals with SCI, (1) the presented setup showed good to excellent test–retest reliability for all extracted gait parameters, (2) individuals with SCI show consistent gait profiles between two measurements within 1 week, and (3) the sensor setup is suitable to detect patient-specific gait characteristics and distinguish patient-specific gait impairments.

### Test–retest reliability of spatiotemporal parameters

It has been demonstrated previously that spatiotemporal gait parameters assessed with IMUs show high validity and good test–retest reliability in individuals with Parkinson’s disease^16^, multiple sclerosis^17^, and stroke^18^ with mild gait impairments. Typically, the algorithms used to calculate spatiotemporal parameters rely on detecting gait events such as heel strike or foot off. Different methods are used to detect these events, and based on their complexity, they show different robustness against individual differences and disturbed gait^32^. Typically, the robustness of gait event detection algorithms decreases with poorer walking ability of an individual, as they usually rely on fixed thresholds, which makes them prone to fail in poor walkers^20^. The algorithm used in this study uses adaptive thresholds to detect each individual’s gait cycles and gait events^20^. This approach resulted in good to excellent reliability (ICC: 0.77–0.99) of spatiotemporal and dynamic parameters, even in individuals with severe gait impairments. ICCs were higher than 0.75 for all parameters, corresponding to good to excellent reliability^29^. It is known that the walking protocols used to collect the gait data have an influence on its reliability^6^. Walking speed and cadence for instance can be reliably assessed over 10 m in SCI^33^, therefore requiring only a few steps to be accurate. However, a higher number of steps is needed to assess dynamic parameters reliably. In elderly and those with Parkinson’s disease, continuous walking protocols with at least 15 strides are recommended to assess reliable dynamic parameters^34,35^. We used a continuous walking protocol (2mWT) that resulted in assessments of at least 79 strides per individual, allowing for a detailed and reliable analysis of different aspects of walking function. To further increase the reliability of spatiotemporal and especially dynamic parameters, the IMU-based assessment could also be done during a 6-min walk test, which is also considered to be reliable in SCI^36^. However, longer testing protocols and the related exhaustion should be avoided in the case of poor walkers.

### Consistency of gait profiles

Recovery of gait is of high relevance for individuals with incomplete SCI^37^. Restoration of gait consists of improvements in functional (e.g. speed, endurance) as well as qualitative (e.g. kinematic, biomechanical) aspects^38^. However, typical clinical measures such as timed measures only assess functional gait recovery and do not inform about movement quality. This is a limitation for clinical trials in neurological disorders, as outcome measures focusing purely on functional gait recovery can miss important information on gait recovery or disease progression. For example, it has been shown that variability parameters for step time and stance time are able to distinguish between healthy controls and individuals with MS in the absence of clinical gait impairments as determined by the EDSS^39^. Also, for fall prediction, spatiotemporal and dynamic parameters are more sensitive than functional outcomes like walking speed^40^. This highlights the potential of sensor-derived gait profiles as a sensitive tool to characterise and monitor gait parameters in individuals with gait impairments. We could demonstrate that gait profiles assessed with IMUs were reliable in SCI, which is a prerequisite for their clinical implementation. In addition, the derived gait profiles were able to reveal different gait patterns in individuals with comparable walking performance. Studies in stroke survivors have identified several altered kinematic gait patterns including decreased knee flexion and decreased dorsiflexion during the swing phase compared to healthy controls. In order to achieve sufficient foot clearance despite these kinematic alterations, patients often develop compensation strategies (e.g., pelvic hiking and circumduction)^41^. Our data shows that individuals with SCI with a similar 2mWT performance can also utilize different gait strategies. For example, Fig. 3B illustrates that subject 02 uses more compensatory movements (reflected in a more asymmetrical gait pattern) than subject 13. This can be explained by the profound left–right lower limb muscle strength asymmetry (assessed by the LEMS) of subject 02, while subject 13 has identical (maximal) muscle strength in both legs. Thus, IMU-based gait analysis allows us to identify patient-specific deficits that cannot be determined by a simple timed walking test. This is of high relevance for longitudinal assessments in SCI. Measurements of gait over time after injury can inform us about primary adaptations in motor behaviour as response to injury and, over time, demonstrate mechanisms of improvement (i.e., compensation vs regeneration).

It has been shown in individuals with a neurological disease that the walking speed differs when measured in clinical vs community environments^42^. These results align with a study performed with individuals with Parkinson’s disease, where a significant difference has been found for all gait parameters between laboratory and free-living environments^10^. Therefore, it is crucial to move the assessment of gait out of the laboratory and into more real-life environments such as at home and in the community. IMUs allow to capture gait over extended periods and can help to provide a more comprehensive picture of individuals’ gait deficits^6^. However, the automatic recognition of walking bouts during everyday life remains challenging, especially for patients with a distinct pathological gait pattern. Further, the threshold for determining the minimum number of steps required to classify an activity as walking remains unknown^9^. IMUs could be used in large, multicentre, cohort, and longitudinal studies, as well as in trials investigating different treatments, where they can facilitate the collection and improve the quality of gait data.

### Limitations

A limitation of this study is the absence of reference data from healthy individuals, which hinders the ability to compare and quantify the pathological nature of gait patterns in individuals with SCI. Given that the primary objective of this study was to assess reliability and sensitivity of gait parameters in detecting inter-individual differences, reference data collection from healthy individuals was not pursued. However, acquiring such data will be the next crucial step towards enabling the quantification of the extent of pathology in the gait patterns of individuals with spinal cord injuries.

It's worth noting that the z-scores were computed to visualize the spatiotemporal parameters among individuals with SCI and to highlight the potential of sensor-derived parameters in identifying distinct walking strategies. However, the calculated z-scores should not be used to compare different spatiotemporal parameters against each other, as normal distribution was not given in all scores.

## Conclusion

This study demonstrated good to excellent test–retest reliability for all gait parameters of an IMU-based gait analysis in individuals with SCI. Based on these findings, IMU-based gait analysis is clinically feasible (simple handling, time effective) to complement gait assessments while providing detailed and objective measures of gait characteristics and changes over time.

### Supplementary Information

Supplementary Figure 1.

Supplementary Table 1.

Supplementary Table 2.

## Data Availability

The data sets collected and analysed as part of the current study are available from the corresponding author upon reasonable request.

## References

[1] Mansour JM, Pereira JM (1987). Quantitative functional anatomy of the lower limb with application to human gait. J. Biomech..

[2] Stolze H (2005). Prevalence of gait disorders in hospitalized neurological patients. Mov. Disord..

[3] Moon Y, Sung JH, An R, Hernandez ME, Sosnoff JJ (2016). Gait variability in people with neurological disorders: A systematic review and meta-analysis. Hum. Mov. Sci..

[4] Schuna JM, Tudor-Locke C (2012). Step by step: Accumulated knowledge and future directions of step-defined ambulatory activity. Res. Exerc. Epidemiol..

[5] Chaudhuri A, Behan PO (2004). Fatigue in neurological disorders. Lancet.

[6] Lord S, Galna B, Rochester L (2013). Moving forward on gait measurement: Toward a more refined approach. Mov. Disord..

[7] Dietz V, Fouad K (2014). Restoration of sensorimotor functions after spinal cord injury. Brain.

[8] Rossignol S, Frigon A (2011). Recovery of locomotion after spinal cord injury: Some facts and mechanisms. Annu. Rev. Neurosci..

[9] Carcreff L (2020). Comparison of gait characteristics between clinical and daily life settings in children with cerebral palsy. Sci. Rep..

[10] Del Din S, Godfrey A, Galna B, Lord S, Rochester L (2016). Free-living gait characteristics in ageing and Parkinson’s disease: Impact of environment and ambulatory bout length. J. Neuroeng. Rehabil..

[11] Rehman RZU (2019). Selecting clinically relevant gait characteristics for classification of early Parkinson’s disease: A comprehensive machine learning approach. Sci. Rep..

[12] Ancillao A (2016). Analysis and measurement of human motion: Modern protocols and clinical considerations. J. Robot. Mech. Eng. Res..

[13] Iosa M, Picerno P, Paolucci S, Morone G (2016). Wearable inertial sensors for human movement analysis. Expert Rev. Med. Devices.

[14] Picerno P (2017). 25 years of lower limb joint kinematics by using inertial and magnetic sensors: A review of methodological approaches. Gait Posture.

[15] Mansour KB, Rezzoug N, Gorce P (2015). Analysis of several methods and inertial sensors locations to assess gait parameters in able-bodied subjects. Gait Posture.

[16] Jakob V (2021). Validation of a sensor-based gait analysis system with a gold-standard motion capture system in patients with Parkinson’s disease. Sensors.

[17] Flachenecker F (2019). Objective sensor-based gait measures reflect motor impairment in multiple sclerosis patients: Reliability and clinical validation of a wearable sensor device. Mult. Scler. Relat. Disord..

[18] Lefeber N, Degelaen M, Truyers C, Safin I, Beckwee D (2019). Validity and reproducibility of inertial physilog sensors for spatiotemporal gait analysis in patients with stroke. IEEE Trans. Neural Syst. Rehabil. Eng..

[19] Treacy D (2017). Validity of different activity monitors to count steps in an inpatient rehabilitation setting. Phys. Ther..

[20] Werner C, Easthope CA, Curt A, Demkó L (2021). Towards a mobile gait analysis for patients with a spinal cord injury: A robust algorithm validated for slow walking speeds. Sensors.

[21] Popp WL (2019). Wearable sensors in ambulatory individuals with a spinal cord injury: From energy expenditure estimation to activity recommendations. Front. Neurol..

[22] Balasubramanian S, Melendez-Calderon A, Roby-Brami A, Burdet E (2015). On the analysis of movement smoothness. J. Neuroeng. Rehabil..

[23] Vagenas GK, Hoshizaki B (1992). A multivariable analysis of lower extremity kinematic asymmetry in running. Int. J. Sport Biomech..

[24] Lord S (2013). Independent domains of gait in older adults and associated motor and nonmotor attributes: Validation of a factor analysis approach. J. Gerontol. Ser. A Biol. Sci. Med. Sci..

[25] Mansour KB, Gorce P, Rezzoug N (2017). The multifeature gait score: An accurate way to assess gait quality. PLoS ONE.

[26] Enright PL (2003). The six-minute walk test. Respir. Care.

[27] Colyer SL, Evans M, Cosker DP, Salo AIT (2018). A review of the evolution of vision-based motion analysis and the integration of advanced computer vision methods towards developing a markerless system. Sports Med. Open.

[28] Koo TK, Li MY (2016). A guideline of selecting and reporting intraclass correlation coefficients for reliability research. J. Chiropr. Med..

[29] Koo TK, Li MY (2016). A guideline of selecting and reporting intraclass correlation coefficients for reliability research. J. Chiropr. Med..

[30] Atkinson G, Nevill AM (1998). Statistical methods for assessing measurement error (reliability) in variables relevant to sports medicine. Sports Med..

[31] Flansbjer UB, Holmbäck AM, Downham D, Patten C, Lexell J (2005). Reliability of gait performance tests in men and women with hemiparesis after stroke. J. Rehabil. Med..

[32] Pacini Panebianco G, Bisi MC, Stagni R, Fantozzi S (2018). Analysis of the performance of 17 algorithms from a systematic review: Influence of sensor position, analysed variable and computational approach in gait timing estimation from IMU measurements. Gait Posture.

[33] Bowden MG, Behrman AL (2007). Step activity monitor: Accuracy and test–retest reliability in persons with incomplete spinal cord injury. J. Rehabil. Res. Dev..

[34] Galna B, Lord S, Rochester L (2013). Is gait variability reliable in older adults and Parkinson’s disease? Towards an optimal testing protocol. Gait Posture.

[35] Kroneberg D (2019). Less is more—Estimation of the number of strides required to assess gait variability in spatially confined settings. Front. Aging Neurosci..

[36] Scivoletto G (2011). Validity and reliability of the 10-m walk test and the 6-min walk test in spinal cord injury patients. Spinal Cord..

[37] Anderson KD (2004). Targeting recovery: Priorities of the spinal cord-injured population. J. Neurotrauma.

[38] Shin SY, Lee RK, Spicer P, Sulzer J (2020). Does kinematic gait quality improve with functional gait recovery? A longitudinal pilot study on early post-stroke individuals. J. Biomech..

[39] Sosnoff JJ, Sandroff BM, Motl RW (2012). Quantifying gait abnormalities in persons with multiple sclerosis with minimal disability. Gait Posture.

[40] Subramaniam S, Faisal AI, Deen MJ (2022). Wearable sensor systems for fall risk assessment: A review. Front. Digit. Health.

[41] Stanhope VA, Knarr BA, Reisman DS, Higginson JS (2014). Frontal plane compensatory strategies associated with self-selected walking speed in individuals post-stroke. Clin. Biomech..

[42] Graham JE, Ostir GV, Fisher SR, Ottenbacher KJ (2008). Assessing walking speed in clinical research: A systematic review. J. Eval. Clin. Pract..

