# Supplementary material for: Reliability of patient-specific gait profiles with inertial measurement units during the 2-min walk test in incomplete spinal cord injury
Source: Sci Rep. 2024 Feb 6;14:3049. doi: 10.1038/s41598-024-53301-y (PMC10847409; doi:10.1038/s41598-024-53301-y)
Supplement: Supplementary file 1 — Supplementary Figure 1. [file 41598_2024_53301_MOESM1_ESM.docx]

**Supplementary figure 1**

**
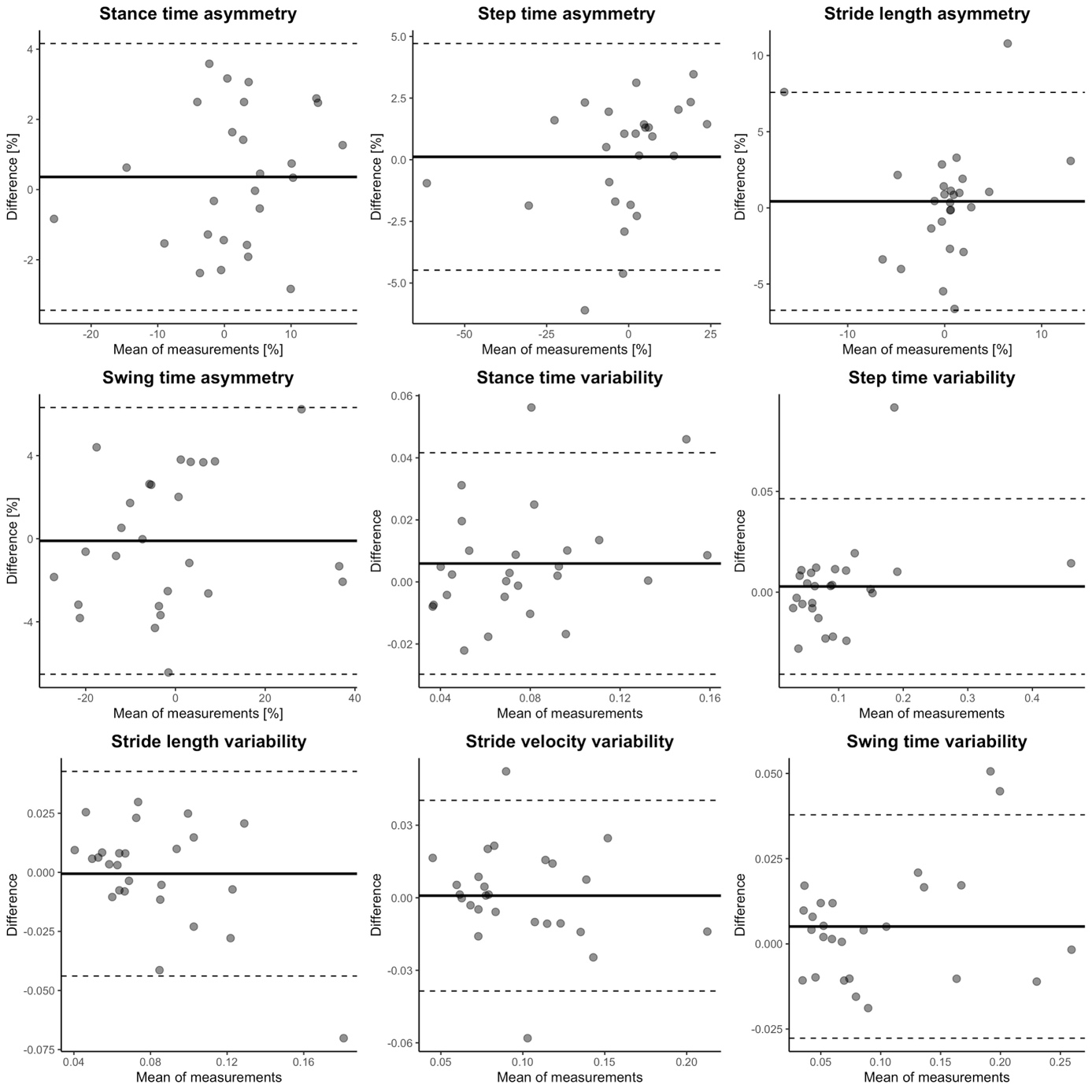
**

Supplementary figure 1: Bland-Altman plots to assess test-retest reliability of asymmetry and variability parameters. The solid lines represent the means and the dashed lines the 95% limits of agreement.
