# Supplementary material for: Reliability of patient-specific gait profiles with inertial measurement units during the 2-min walk test in incomplete spinal cord injury
Source: Sci Rep. 2024 Feb 6;14:3049. doi: 10.1038/s41598-024-53301-y (PMC10847409; doi:10.1038/s41598-024-53301-y)
Supplement: Supplementary file 2 — Supplementary Table 1. [file 41598_2024_53301_MOESM2_ESM.docx]

Supplementary table 1: definition of spatiotemporal gait parameters [19]

| **Parameter** | **Definition** |
| --- | --- |
| Stride velocity | Stride length divided by stride time. |
| Stride time | Duration from one heel strike to the next consecutive heel strike of the same leg. |
| Step time | Duration from one heel strike to the next heel strike on the contralateral leg. |
| Swing time | Duration from foot off till the next heel strike of the same leg. |
| Relative swing time | Percentage of the swing time assuming that the whole gait cycle is 100% (approximately 40% of the gait cycle). |
| Stance time | Duration from heel strike to the next foot off. |
| Relative stance time | Percentage of the stance time assuming that the whole gait cycle is 100% (approximately 60% of the gait cycle). |
| Double support | Time per gait cycle during which both feet are on the ground (from heel strike to the next foot off on the contralateral side, summed up for both legs during a gait cycle). |
| Relative double support | Percentage of the double support assuming that the whole gait cycle is 100%. |
| Stride length | Distance between any two successive heel strikes of the same foot. |
| Vertical foot displacement | Vertical displacement range of the foot during a gait cycle. |
| Horizontal foot displacement | Horizontal displacement range of the foot during a gait cycle. |
| Smoothness | A measure describing the quality of a movement by its continuality, calculated based on analytical models from Balasubramanian et al. [24]. |
