# Supplementary material for: Reliability of patient-specific gait profiles with inertial measurement units during the 2-min walk test in incomplete spinal cord injury
Source: Sci Rep. 2024 Feb 6;14:3049. doi: 10.1038/s41598-024-53301-y (PMC10847409; doi:10.1038/s41598-024-53301-y)
Supplement: Supplementary file 3 — Supplementary Table 2. [file 41598_2024_53301_MOESM3_ESM.docx]

|  |  | **Pace** | | | **Variability** | | | | **Rhythm** | | | **Asymmetry** | | | **Postural control** | |
| --- | --- | --- | --- | --- | --- | --- | --- | --- | --- | --- | --- | --- | --- | --- | --- | --- |
| Sub | 2mWT distance | Stride velocity | Stride length | Swing time variability | Step time variability | Stance time variability | Stride velocity variability | Stride length variability | Step time | Swing time | Stance time | Step  time asymmetry | Swing time asymmetry | Stance time asymmetry | Double support | Stride length asymmetry |
| 1 | 25 | -1.50 | -1.85 | -0.03 | 0.26 | 0.15 | 1.21 | 1.67 | 1.78 | -0.32 | 2.10 | -0.72 | 0.52 | -0.36 | 3.31 | 0.15 |
| 2 | 95 | -0.31 | -0.11 | 1.97 | 0.94 | 2.03 | 0.35 | 0.53 | -0.09 | 0.45 | -0.22 | -1.57 | 2.34 | -2.90 | -0.51 | 2.08 |
| 3 | 95 | -0.23 | -0.08 | 0.39 | 0.06 | 0.29 | -1.13 | -1.73 | -0.19 | 0.13 | -0.25 | 1.09 | -1.10 | 1.13 | -0.35 | 0.29 |
| 4 | 150 | 0.81 | 0.54 | -0.75 | -0.41 | -0.26 | -0.48 | -0.57 | -0.83 | -0.64 | -0.79 | 0.31 | -0.04 | -0.02 | -0.68 | -0.14 |
| 5 | 180 | 1.34 | 0.55 | -0.89 | -0.71 | -1.08 | -0.59 | -0.38 | -1.12 | -1.29 | -0.97 | 0.17 | 0.06 | -0.14 | -0.71 | -0.19 |
| 6 | 80 | -0.45 | -0.10 | -0.49 | -0.16 | -0.28 | 0.21 | 0.29 | 0.17 | 1.07 | -0.08 | -0.09 | 0.33 | -0.64 | -0.14 | 0.70 |
| 7 | 160 | 1.07 | 0.99 | -0.89 | -0.74 | -1.06 | -1.03 | -1.14 | -0.81 | -0.82 | -0.72 | 0.29 | -0.30 | 0.28 | -0.62 | 0.03 |
| 9 | 117 | 0.25 | 0.45 | -0.69 | -0.43 | -0.86 | -0.66 | -0.66 | -0.42 | -0.66 | -0.33 | 0.12 | 0.20 | -0.40 | -0.03 | 0.13 |
| 11 | 158 | 1.00 | 1.23 | -0.74 | -0.59 | -1.06 | -0.63 | -0.49 | -0.65 | -0.77 | -0.56 | 0.26 | -0.32 | 0.24 | -0.45 | 0.01 |
| 12 | 150 | 0.52 | 0.19 | -0.73 | -0.72 | -1.14 | -1.69 | -1.65 | -0.80 | -0.83 | -0.71 | 0.34 | -0.34 | 0.29 | -0.61 | 0.58 |
| 13 | 94 | -0.26 | 0.02 | -0.32 | -0.43 | -0.23 | 0.38 | 0.37 | -0.25 | -0.37 | -0.19 | 0.10 | -0.56 | 0.39 | -0.10 | -0.21 |
| 14 | 241 | 2.54 | 2.30 | -0.91 | -0.53 | -0.49 | -0.79 | -1.11 | -1.04 | -1.07 | -0.92 | 0.40 | -0.02 | 0.02 | -0.75 | 0.45 |
| 17 | 44 | -1.11 | -1.18 | 0.99 | 0.53 | 0.67 | 0.92 | 0.90 | 0.74 | 0.98 | 0.60 | 1.39 | -1.08 | 0.88 | 0.39 | -1.17 |
| 18 | 108 | 0.07 | 0.59 | -0.20 | -0.11 | -0.28 | 0.60 | 1.22 | -0.13 | 0.75 | -0.34 | 0.13 | 0.01 | 0.02 | -0.61 | 0.42 |
| 19 | 133 | 0.53 | 0.79 | -0.62 | -0.28 | -0.28 | 0.82 | 0.28 | -0.50 | -0.44 | -0.46 | -0.31 | 0.37 | -0.47 | -0.42 | -0.15 |
| 20 | 37 | -1.24 | -1.29 | 0.02 | 0.02 | 0.31 | 1.56 | 2.01 | 1.17 | 0.65 | 1.14 | -0.22 | -0.64 | 0.34 | 1.09 | -0.53 |
| 21 | 144 | 0.73 | 1.14 | -1.07 | -0.54 | -0.94 | -0.97 | -1.25 | -0.50 | -0.18 | -0.53 | -0.31 | 0.09 | -0.14 | -0.52 | 0.11 |
| 22 | 97 | -0.15 | 0.27 | 0.84 | -0.13 | 0.66 | -0.81 | -0.77 | -0.05 | 0.53 | -0.19 | 0.88 | -1.47 | 1.63 | -0.30 | 0.07 |
| 23 | 101 | -0.10 | 0.27 | -1.03 | -0.57 | -0.97 | -0.33 | -0.37 | -0.12 | 0.18 | -0.19 | 0.47 | 0.08 | -0.17 | -0.28 | -0.05 |
| 24 | 150 | 0.84 | 0.39 | -0.25 | -0.14 | -0.12 | -0.73 | -0.72 | -0.92 | -0.81 | -0.85 | 0.86 | -0.67 | 0.84 | -0.75 | 0.06 |
| 25 | 41 | -1.20 | -1.22 | 2.33 | 3.88 | 1.43 | 1.01 | 0.23 | 1.15 | 0.37 | 1.14 | -3.34 | 2.41 | -1.81 | 1.03 | 0.75 |
| 26 | 37 | -1.29 | -1.03 | 1.11 | 0.57 | -0.28 | 1.13 | 1.38 | 1.86 | 0.91 | 1.90 | -1.22 | 1.61 | -1.09 | 2.29 | -0.94 |
| 27 | 63 | -0.75 | -1.24 | -0.38 | -0.46 | -0.74 | -0.95 | -0.81 | -0.34 | -0.14 | -0.35 | 0.18 | 0.62 | -0.77 | -0.44 | -0.30 |
| 28 | 126 | 0.39 | 0.28 | -0.84 | -0.70 | -1.24 | -0.82 | -0.72 | -0.64 | -0.80 | -0.55 | -0.01 | 0.20 | -0.33 | -0.41 | -0.03 |
| 29 | 36 | -1.28 | -1.17 | 0.96 | 0.44 | 1.27 | 3.32 | 4.94 | 1.57 | 2.68 | 1.12 | -0.49 | -1.09 | 1.01 | 1.12 | -3.87 |
| 31 | 159 | 1.00 | 0.66 | 0.29 | 0.16 | 0.32 | -0.54 | -0.88 | -0.89 | -1.01 | -0.78 | 1.10 | -1.06 | 1.17 | -0.54 | -0.38 |

Supplementary table 2: Z-scores from day 2

*Z-scores of each subject and each gait parameter from day 2. Blue colour indicates a reduction from the mean and red colour indicates an increase, light colour indicates a deviation of more than 1 standard deviation and dark colour indicates deviation of more than 2 standard deviations.*
